# Insights into a possible role of glucagon-like peptide-1 receptor agonists in the treatment of depression

**DOI:** 10.1007/s43440-021-00274-8

**Published:** 2021-05-18

**Authors:** Jan Detka, Katarzyna Głombik

**Affiliations:** Laboratory of Immunoendocrinology, Department of Experimental Neuroendocrinology, Polish Academy of Sciences, Maj Institute of Pharmacology, 12 Smętna Street, 31-343 Cracow, Poland

**Keywords:** GLP-1, Exendin-4, Liraglutide, Depression, HPA axis, Gut-brain axis

## Abstract

Depression is a highly prevalent mood disorder and one of the major health concerns in modern society. Moreover, it is characterized by a high prevalence of coexistence with many other diseases including metabolic disorders such as type 2 diabetes mellitus (T2DM) and obesity. Currently used antidepressant drugs, which mostly target brain monoaminergic neurotransmission, have limited clinical efficacy. Although the etiology of depression has not been fully elucidated, current scientific data emphasize the role of neurotrophic factors deficiencies, disturbed homeostasis between the nervous system and the immune and endocrine systems, as well as disturbances in brain energy metabolism and dysfunctions in the gut-brain axis as important factors in the pathogenesis of this neuropsychiatric disorder. Therefore, therapeutic options that could work in a way other than classic antidepressants are being sought to increase the effectiveness of the treatment. Interestingly, glucagon-like peptide-1 receptor agonists (GLP-1RAs), used in the treatment of T2DM and obesity, are known to show pro-cognitive and neuroprotective properties, and exert modulatory effects on immune, endocrine and metabolic processes in the central nervous system. This review article discusses the potential antidepressant effects of GLP-1RAs, especially in the context of their action on the processes related to neuroprotection, inflammation, stress response, energy metabolism, gut-brain crosstalk and the stability of the gut microbiota.

**Graphic abstract:**

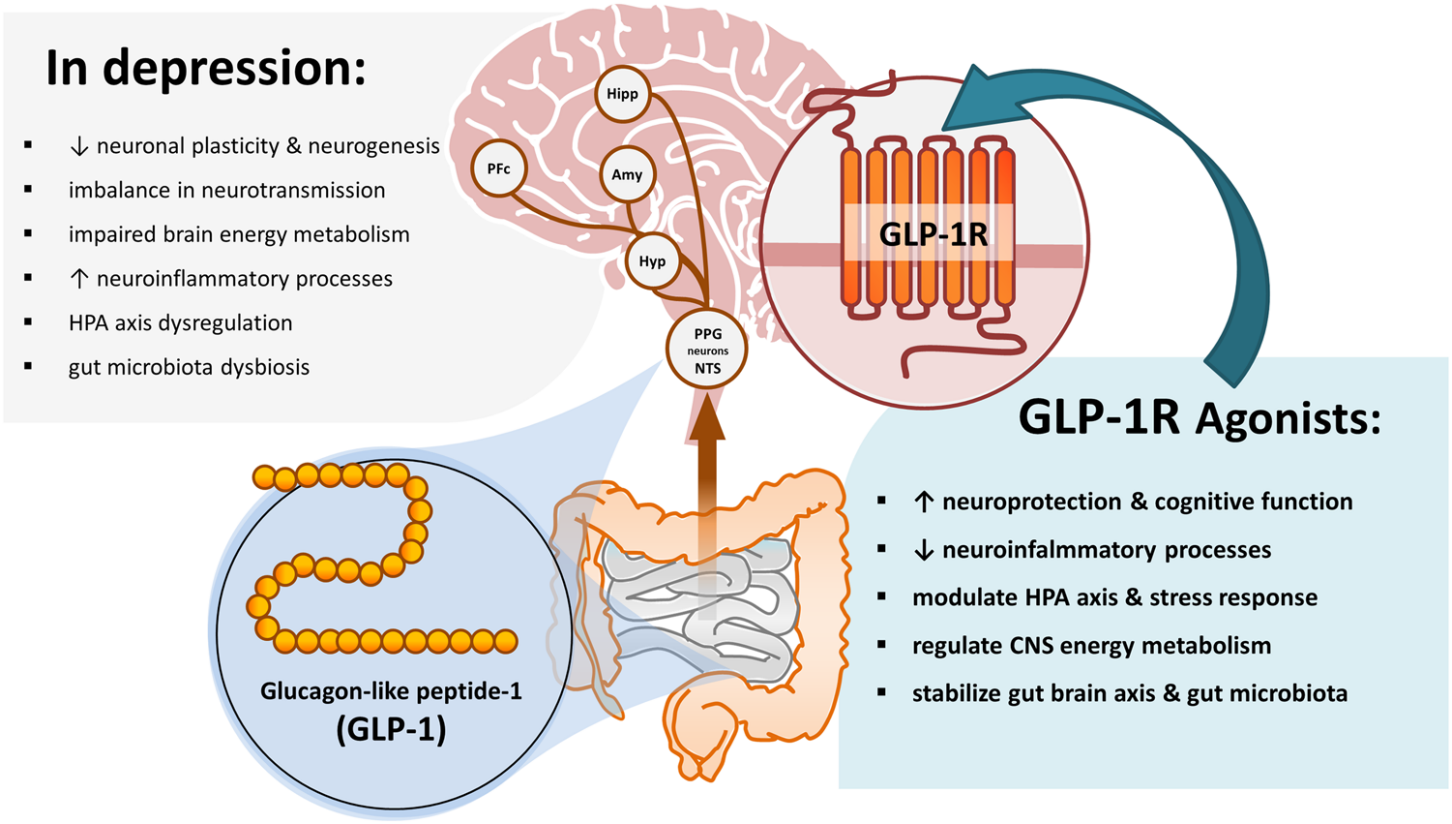

## Introduction

Depression is a chronic and recurrent mood disorder and one of the major health concerns in modern society, which, according to the World Health Organization (WHO), affects more than 264 million people worldwide. It is estimated that 76–85% of people living in low- or middle-income countries and suffering from this mental illness are not adequately treated [1]. Clinical effectiveness of currently used antidepressant drugs, which target monoaminergic systems in the brain mainly by enhancing serotoninergic and noradrenergic neurotransmission, is very often low and insufficient since approximately only one-third of the patients achieve complete remission of depressive symptoms after therapy. Therefore, therapeutic options that could work in a way other than classic antidepressants are being sought to increase the effectiveness of the depression treatment [2].

The limited efficacy of current antidepressant therapies can be explained by the heterogeneity of depressive disorders. Even though extensive research conducted over the last years did not unequivocally establish the etiology of depression, it revealed the complexity of its pathogenesis. In addition to blunted monoaminergic neurotransmission, it was also shown that deficiency in neurotrophic factor effects, disturbances in neurogenesis and synaptogenesis, chronic neuroinflammatory processes, and dysregulation of the hypothalamic–pituitary–adrenal (HPA) axis can largely contribute to the manifestation of depressive symptoms in patients [3–5].

Many recent studies also focus on the metabolic aspects of depression, suggesting the involvement of peripheral and central impairments in energy homeostasis, mitochondrial dysfunction, as well as disturbances in the gut-brain axis and gut microbiota dysbiosis as noteworthy factors in the pathogenesis of depression [6–9]. Moreover, depression is characterized by high prevalence of coexistence with many metabolic diseases, such as type 2 diabetes mellitus (T2DM) and obesity, and brain insulin resistance observed in both of these diseases is currently considered an important factor which may lead to the manifestation of depressive symptoms [10, 11].

Glucagon-like peptide-1 (GLP-1) is a glucoregulatory hormone synthetized in the intestines and the brain, involved in the maintenance of energy homeostasis mainly by stimulating insulin secretion and regulation of appetite. GLP-1 exerts its biological effects by binding to a specific receptor (GLP-1R), which is expressed in different types of peripheral tissues and in the central nervous system [12]. Studies on insulinotrophic and anorexigenic effects of GLP-1 only recently have led to the development of a new class of drugs, often referred to as incretin drugs, which act as GLP-1 receptor agonists (GLP-1RAs) and are currently implemented in therapy for T2DM [13]. Lately, a GLP-1R agonist, liraglutide, has also been approved by the FDA for the treatment of adolescent obesity [14].

Recently, in addition to the central maintenance of food intake, GLP-1R signaling has begun to be considered in the context of the regulation of the stress response, as it is indicated that it can activate both the hypothalamic–pituitary–adrenal (HPA) axis as well as the sympathetic nervous system [15, 16]. Numerous reports also emphasize the neuroprotective role of GLP-1 mediated signaling, as it was proven to protect synapses, improve synaptic functions, enhance neurogenesis, protect neurons from oxidative stress, and reduce neuronal apoptosis. Therefore, GLP-1R agonists are currently being intensively studied for their use in the treatment of neurodegenerative diseases such as Alzheimer’s disease and Parkinson’s disease [17, 18]. Moreover, there are a few studies which indicate that these compounds may also prove useful in the treatment of neuropsychiatric disorders including depression [19, 20].

This review article aims at discussing the potential of therapeutic GLP-1 receptor agonists in alleviating depressive symptoms in the context of modern theories surrounding the pathogenesis of this disease and explores their influence on cognitive processes, neurotransmission, modulation of neuroinflammatory processes, effects on HPA axis activity, brain energy balance, and the functioning of the gut-brain axis.

## Physiology of peripheral and central GLP-1 signaling

GLP-1 is a 30-amino acid peptide synthetized by enteroendocrine L cells of the small and large intestine, and also in the brain primarily by neurons of the nucleus tractus solitarius (NTS) within the caudal brainstem as a product of tissue-specific posttranslational cleavage of a larger precursor molecule preproglucagon (PPG). In the periphery, GLP-1 is released from the gut in response to the presence of food nutrients. Its main functions include stimulation of insulin synthesis and secretion from pancreatic β cells, enhancement of the sensitivity of target organs to insulin, inhibition of glucagon synthesis, and slowing gastric emptying. At the same time, GLP-1 can also enhance the proliferation and differentiation of β cells and protect them from apoptosis [21].

Glucagon-like peptide-1 receptor (GLP-1R) belongs to the family B of G-protein coupled receptors (GPCR). This relatively small GPCR family is characterized by a long extracellular ligand-binding domain stabilized by three disulfide bridges formed by six highly conserved cysteine residues, seven α-helical transmembrane domains, universal for all GPCRs and an intracellular C-terminal domain, responsible mostly for receptor internalization due to the presence of multiple phosphorylation sites [22, 23]. Intracellular signaling, followed by the activation of GLP-1Rs, is mostly mediated through the Gα_s_ subunit and the activation of adenylate cyclase, which increases the levels of cyclic adenosine monophosphate (cAMP). This, in turn, stimulates downstream signaling elements dependent on both protein kinase A (PKA) and exchange protein directly activated by cAMP (EPAC) activation, which triggers the opening of voltage-dependent calcium channels (VDCCs), resulting in insulin exocytosis [24, 25]. However, some studies show that the activation of GLP-1Rs can also initiate different intracellular pathways mediated by Gα_q_ and Gα_i_ [26]. GLP-1Rs are expressed in the pancreas, stomach, intestines, heart, kidneys, lungs as well as multiple regions of the brain [27–29].

Activation of GLP-1 signaling in the brain is mostly triggered by its peripheral release. Upon its secretion from the gut, GLP-1 activates GLP-1Rs located on the vagus nerve afferents, which leads to vagal glutamatergic excitation of PPG expressing neurons in the NTS [30]. The role of GLP-1 in the nervous system largely, but not exclusively, complements peripheral actions of this peptide and encompasses suppression of food and water intake and the reduction of appetite and food reward. However, this anorexigenic action of GLP-1 is maintained through a complex neural circuitry and involves multiple brain areas [25, 31]. Preproglucagon expressing neurons in the NTS project to many parts of the brain, including the hypothalamus, thalamus, parabrachial nucleus, parts of the mesolimbic dopamine system, raphe nucleus, amygdala, hippocampus and medial prefrontal cortex [32]. Distribution of GLP-1Rs in the brain is highly conserved in different mammalian species and pretty much correlates with the projection pattern of PPG-expressing neurons [33]. Suppression of food and water intake by GLP-1 mainly involves interactions with various hypothalamic nuclei, particularly the arcuate and paraventricular nucleus, as well as some hindbrain structures such as the parabrachial nucleus [31].

Interestingly, GLP-1 is also known to decrease the rewarding effects of food intake by interacting with parts of the mesolimbic dopamine system such as the ventral tegmental area (VTA), nucleus accumbens (NAc) [34], and also GABAergic circuits in the laterodorsal tegmental nucleus (LDTg) [35], which makes brain GLP-1R signaling a promising target for the treatment of alcohol and cocaine abuse disorders [36, 37].

## Characteristics of therapeutic GLP-1R agonists

Endogenous GLP-1 is characterized by a very short half-life of only few minutes, as it is quickly degraded by dipeptidyl peptidase-4 (DPP4), an enzyme expressed in many different types of tissues. All of the five therapeutic GLP-1RAs, currently approved for therapy of type 2 diabetes, are peptide molecules, which are administered in subcutaneous injections. They were developed on the basis of either human GLP-1 or exendin-4, a 39-amino acid peptide isolated from the Gila monster (*Heloderma suspectum*) venom, which shares 53% homology with GLP-1 [38]. These molecules are more resistant to degradation by DPP-4, which results in a prolonged half-life in comparison with endogenous GLP-1. However, because of the structural differences among different therapeutic GLP-1RAs, there is also a significant distinction in their stability, which affects their administration schedule, and thus they are often classified as either short- or long-acting. Short-acting GLP-1RAs, which present a half-life of 3–4 h, include exenatide, administered twice daily, and lixisenatide, administered once daily, are both exendin-4 derivatives. Long-acting GLP-1RAs are synthetic peptide molecules with modifications which further improve their stability. For example, liraglutide and semaglutide have the attachments of fatty acid residues, which enable their reversible binding to albumin, while drugs such as dulaglutide and albiglutide are recombinant fusion proteins containing human immunoglobulin G or albumin chains [13]. Both short- and long-acting GLP-1RAs such as exenatide, lixisenatide and liraglutide were shown to effectively cross the blood–brain barrier (BBB) [39–41]. Semaglutide on the other hand displays limited efficacy in crossing the BBB, but despite this fact it was proven to access the brainstem, septal nucleus and hypothalamus [42].

## GLP-1R signaling in the brain exerts antidepressive effects by improving cognitive processes, promoting neuroprotection and neurotransmitter release

Patients suffering from depression are characterized by impaired neuronal activity and synaptic plasticity dysfunction, resulting in volumetric changes in brain regions which regulate emotions or the stress response such as the hippocampus and the prefrontal cortex. The hippocampus, a brain structure associated with memory formation, seems to be particularly affected in depression [43, 44]. Considering the relationship between GLP-1 signaling and behavioral symptoms characteristic of depression, it was shown that intracerebroventricular (*icv*) administration of both GLP-1 and exendin-4 enhances associative and spatial learning, while these effects are inhibited by a GLP-1R antagonist. It is noteworthy that a behavioral analysis of Glp1r +/+ , Glp1r +/− and Glp1r−/− mouse phenotypes and hippocampal Glp1r gene transfer demonstrated that deficiency of this receptor results in decrements in the acquisition of associative contextual learning which can be improved by hippocampal Glp1r somatic cell gene transfer [45]. Recent data have indicated that such an effect may depend on the influence on metabolic processes because in chronic unpredictable mild stress (CUMS) in mice, the decreased GLP-1 level in the hippocampus was increased with metformin treatment [46]. Metformin has been found to produce antidepressive effects by enhancing the responsiveness of the serotoninergic neurons in the dorsal raphe [47], but the main impact of GLP-1 is exerted by AMP-activated protein kinase (AMPK), a major cellular regulator of lipid and glucose metabolism [48]. Liraglutide administration weakened depressive- and anxiety-like behaviors in a depression mouse model of chronic administration of corticosterone and attenuated hyperactivity induced by this steroid. Additionally, chronic liraglutide treatment protected synaptic plasticity impairments and, in contrast to fluoxetine, attenuated the inhibition of long-term potentiation in the hippocampus, which was induced by corticosterone in 30-day treatment [49]. What is more, it seems that GLP-1R agonists may be useful as an adjunctive to traditional therapy to treat cognitive impairments observed in the course of depression, especially in women. Studies on mice demonstrated that administration of exendin-4 (*ip*) prior to cognitive tests enhanced performance during difficult probe tests but only in females [50].

For a long time, the manifestation of depressive symptoms has been also associated with the deficits in neurotrophic factor action in the brain [51], especially highlighting the role of diminished brain-derived neurotrophic factor (BDNF) signaling as important for the pathogenesis of this mental disorder [52]. GLP-1R agonists in animal models of many neurodegenerative diseases repeatedly increased the expression of BDNF in the brain. For example, a novel dual agonist of GLP-1/GIP receptors significantly increased BDNF levels in a mouse model of Parkinson’s disease, induced by 1-methyl-4-phenyl-1,2,3,6-tetrahydropyridine (MPTP) administration [53]. Recently, an increase in BDNF synthesis has been demonstrated in a pentylenetetrazol (PTZ) kindling model of epilepsy, after chronic treatment with liraglutide in combination with antiepileptic drug, levetiracetam, which also exerted antidepressive effects, e.g. by decreasing immobility time in the forced swim test [54].

On the other hand, in addition to the processes described above, it has been well established that neurotransmitters play a key role in behavior regulation and this is a clear relationship between their imbalance and specific symptoms of a major depressive disorder [55]. Data have shown that an *icv* injection of GLP-1 diminished the level of 5-HT in the hypothalamus of Sprague–Dawley rats compared with control animals. Moreover, administration of exendin-4 led to a reduction in 5-HT and 5-HIAA, whereas GLP-1R antagonists increased the levels of serotonin and its metabolites [56]. Furthermore, it was demonstrated that GLP-1 receptor plays a regulatory role in the hippocampal function and GLP-1 receptor agonists enhanced GABAA signaling through pre-and postsynaptic mechanisms in rats [57], while exendin-4 therapy normalized cognitive dysfunction and improved impaired hippocampal synaptic plasticity in a mice model of diet-induced obesity [58]. Summing up, it seems that the role of the GLP-1 pathway in the regulation of neurotransmission in the central nervous system cannot be omitted when assessing the influence of this factor on changes observed in depression.

## GLP-1R agonists may exert antidepressant effects by modulating inflammatory processes in the brain

Chronic inflammatory processes in the brain associated with excessive activation of microglia and increased production of pro-inflammatory cytokines are one of the key factors leading to the development of depressive symptoms, as patients suffering from this mental illness are very often characterized by elevated levels of tumor necrosis factor-alpha (TNF-α), interleukin-6 (IL-6), interleukin-1 beta (IL-1β), C-reactive protein (CRP), and interferon-gamma (IFN-γ) in the cerebrospinal fluid and the blood [59–61].

GLP-1 as well as its stable therapeutic analogs were shown to decrease the levels of inflammatory markers in peripheral tissues and brain cells. For example, exendin-4 at the concentration of 50 nM downregulated the expression of NF-κB p65 in isolated human pancreatic β cells [62]. Moreover, treatment with GLP-1 resulted in a decrease of lipopolysaccharide (LPS)-induced activation of the BV-2 microglial cell line, accompanied by a decrease in TNF-α secretion [63]. In a recent study by Shan et al. [64], which involved a co-culture of primary astrocytes with the bEnd.3 endothelial cell line, incubation with exendin-4 reduced the levels of the astrocyte-derived vascular endothelial growth factor (VEGF-A), matrix metalloproteinase-9 (MMP-9), chemokine monocyte chemoattractant protein-1 (MCP-1), and the chemokine C-X-C motif ligand 1 (CXCL-1), induced by oxygen–glucose deprivation (OGD), and subsequently led to the increased expression of tight junction proteins in the bEnd.3 cells. These results may suggest that GLP-1R agonists may alleviate the blood–brain barrier breakdown induced by inflammatory factors, which is sometimes observed in depression [65].

Neuroprotective properties of GLP-1RAs observed in animal models of neurodegenerative diseases such as Alzheimer’s disease are very often associated with the reduction of inflammatory processes in the central nervous system. In APP/PS1 mice, the cognitive improvement observed after *ip* administration of liraglutide was accompanied by decreased activation of microglia [66]. In the light of the current research, it is not known whether GLP-1RAs can also reduce the inflammatory component in animal models of depression. In one study, it was demonstrated that exendin-4 prevented LPS-induced depressive-like symptoms in rats and displayed antidepressant-like properties measured in the forced swim test, but this effect was not mediated by the modulation of neuroinflammatory processes, since the levels of IL-1β and TNF-α were not affected by exendin-4 treatment [67]. Thus, further studies are needed to better elucidate the effects of different GLP-1R agonists on anti-inflammatory actions in depression.

## The role of brain GLP-1 signaling in the modulation of the stress response and anxiety-like behavior and its association with depression

Under a stress stimulus, the HPA axis becomes activated which results in the release of glucocorticoids [68]. It is known that the acute HPA response to stress plays an important role and has numerous functions which promote cells survival, but chronic activation of this axis by stressful episodes may lead to a wide range of pathological conditions, including depression. It is estimated that nearly 60% of depressive episodes are preceded by an exposure to various types of stressors [69]. Depressed patients are often characterized by elevated blood cortisol levels and changes in the circadian secretion of this hormone, an increased number of corticotropin-releasing hormone (CRH)-expressing neurons in the hypothalamic paraventricular nucleus (PVN), as well as an impairment in a negative feedback mechanism, which can be measured in the dexamethasone suppression test [70, 71].

The role of GLP-1 in the regulation of the stress axis seems to be undeniable. GLP-1 immunoreactive fibers and nerve terminals are distributed in the parvocellular parts of the PVN and innervate this structure, directly connecting GLP-1 with the CRH neurons [72]. Direct influence of GLP-1R agonists on CRH expression was also observed in both an in vitro cellular model [73] and experimental animals [74]. It was demonstrated that GLP-1 brain administration elevated the circulating concentrations of the adrenocorticotropic hormone (ACTH) and corticosterone and also enhanced anxiety in behavioral measurements [75]. The stimulatory effects of GLP-1 on the HPA axis were not limited to rodents; increased cortisol levels were observed in both healthy humans and type 1 diabetes patients to whom GLP-1 had been administered peripherally [76]. Recent findings from studies on humans and animals demonstrate that GLP-1R agonists can also modulate the stress response via HPA axis activation but data obtained so far are ambiguous. These data showed that subchronic (7 to 14 days) exendin-4 or liraglutide administration in rodents resulted in hyperactivation of the HPA, disruption of glucocorticoid circadian secretion, hypertrophy of the adrenal gland, and dysregulation of pituitary-adrenal stress responses [77]. Conversely, in a recent randomized, double-blind, placebo-controlled study involving twenty healthy volunteers, a once-weekly injection of a GLP-1R agonist, dulaglutide, for three weeks did not influence HPA axis activity, showing no significant effects on circadian rhythms of blood or salivary cortisol secretion, and the level of this hormone also was not changed after the dexamethasone suppression test in comparison with the placebo-receiving group [78]. However, at this point it should be noted that different incretin drugs are characterized by different pharmacokinetics, and therefore, they are taken by patients at different time intervals. Thus, they may have a different effect on the activity of the HPA axis in humans.

There are also some data suggesting that excessive glucocorticoid action under stress can influence the endogenous GLP-1 system. Recent *post mortem* studies revealed that GLP-1R expression was downregulated in the dorsal lateral prefrontal cortex and hippocampus of patients suffering from mood disorders, and those changes further correlated with an elevated body mass index (BMI) [79]. In animal studies, administration of dexamethasone caused a reduction of GLP-1 secretion from the intestinal L cells [80] and also decreased the circulating levels of this peptide, which led to glucose intolerance and peripheral insulin resistance in rats [81]. In other experiments, acute stress also caused rapid downregulation of preproglucagon gene expression in the NTS neurons in rat brains [82]. In our studies conducted in a prenatal stress model (PS) of depression in rats, adult PS animals showed a significant decrease in GLP-1R protein levels in the hippocampus after the acute immobility stress [83]. The concentration of this receptor was also reduced in the hippocampus of prenatally stressed rats subsequent to a 12-week high-fat diet [84]. Taken together, these results may suggest that GLP-1 is an important mediator of the stress response in the brain. It is also noteworthy that central GLP-1R signaling appears to be impaired in chronic stress conditions or under the influence of a high concentration of glucocorticoids, often observed in patients with depression.

Depression is a disorder which often coexists with anxiety or significant anxiety symptoms [85]. GLP-1 has been tested as a regulator of anxiety behavior and both GLP-1 and exendin-4 (administered *ip*, into the lateral ventricle or intra-dorsal raphe) induced anxiety-like behavior, which was measured in the black and white box, elevated plus maze and open field tests in Spraque-Dawley rats [86]. Nonetheless, the neural base of anxiety from which GLP-1 exerts its anxiogenic effect remains unclear. The supramammillary nucleus, located in the hypothalamus, is a new candidate that appears to be responsible for GLP-1 mediated anxiety effects because selective activation of GLP-1Rs in this nucleus by exendin-4 resulted in intensified anxiety-like behavior both in male and female rats. Interestingly, selective knockdown of GLP-1Rs in the supramammillary nucleus led to anxiolytic response, which was observed only in females [87]. It is known that women are diagnosed with an anxiety disorder twice as likely as men, and the GLP-1 pathway may be involved in this dependence.

## GLP-1R signaling regulates brain energy homeostasis and may improve mitochondrial function in depression

In addition to the disturbed neuroimmune and neuroendocrine crosstalk, disruptions in brain metabolism and mitochondrial functions seem to be an emergent field of interest in the context of depression pathogenesis. Alterations in mitochondrial functions such as oxidative phosphorylation (OXPHOS) and membrane polarity, which enhance oxidative stress and apoptosis, may precede the development of depressive symptoms [84, 88, 89]. Growing body of evidence suggests that dysfunction of the GLP-1 pathway, which directly targets mitochondria and preserves mitochondrial functions, contributes to the pathophysiology of depression [86]. As an insulinotropic hormone, GLP-1 plays a substantial role in the regulation of glucose homeostasis, not only in the periphery but also in the central nervous system [90]. On the other hand, disturbances observed in the course of depression do not clearly indicate whether they are the effects of disruption of metabolic processes in the brain. Nevertheless, it is known that in a normal in vivo state, glucose is the only significant metabolism substrate in the brain which determines the proper functions of neurons. Thus, metabolic changes may be key players in many disorders, including depression [91, 92].

In animal models of neurodegenerative diseases, it has been demonstrated that administration of GLP-1RAs can result in the improvement of mitochondrial stability. Pretreatment with liraglutide (25 nmol/kg; *ip*) for 7 days resulted in the alleviation of mitochondrial stress induced by status epilepticus in the hippocampus of Sprague–Dawley rats [93]. In a focal cerebral cortical ischemic mouse model, liraglutide improved motor functional recovery and promoted axonal sprouting by restoring the activities of several Krebs cycle enzymes, i.e. isocitrate dehydrogenase, α-ketoglutarate dehydrogenase, and succinate dehydrogenase, and at the same time reduced generation of reactive oxygen species stabilized the mitochondrial membrane potential, enhanced the levels of ATP, and enhanced the activity of the mitochondrial complex I [94].

Most studies of central glucose homeostasis have focused on neurons. However, recent data also suggest that astrocytes may be involved in systemic energy balance control. These brain cells rely on glycolysis in energy generation and produce lactate, which serves as both main energy fuel and a signaling molecule for neurons. It was demonstrated, that metabolic disturbances in hypothalamic astrocytes, caused either by selective knock-out of insulin receptors or decreased generation of reactive oxygen species (ROS) modulated glucose transport via BBB and also led to modification in behavioral responses in experimental animals [95, 96]. On the other hand, GLP-1R signaling in astrocytes was proven to be essential for maintaining mitochondrial integrity and function of the hypothalamic astrocytes, as deletion of GLP-1Rs resulted in the adaptive cellular stress response with the improvement of glucose metabolism and memory formation [97].

## GLP-1R agonists may improve the function of the gut-brain axis and stability of the gut microbiota

Many recent reports also emphasize the role of the gut-brain axis and stability of the gut microbiota in the development of mood disorders, including depression. The gastrointestinal tract and the brain are involved in a complex bidirectional communication, which is mediated by the endocrine, immune and neural pathways, involving a plethora of different neurotransmitters, cytokines and peptide hormones [98]. People with depression are often characterized by a significantly lower number of microorganisms in the GI tract along with a decreased diversity of the gut microbiome [99]. In the light of the current research, it is speculated that the maladaptive changes in the gut microbiota can influence the central nervous system and evoke depressive symptoms, and the development of depression can influence gut microbiome stability, and both of these hypotheses appear to be supported by the results of human and animal studies [100]. For example, in the studies carried out by Kelly et al. [99], which involved transplantations of fecal microbiota obtained from depressed patients into a microbiota-depleted antibiotic rat model, the animals developed depressive-like symptoms, which were manifested by behavioral differences such as decreased sucrose intake in the sucrose preference test, a lower number of entries to an open arm in the elevated plus-maze test, and an increase in a plasma kynurenine/tryptophan ratio. Similar results were observed in an experiment involving a germ-free mouse strain, where the animals transplanted with microbiome isolated from depressed patients showed an increase in immobility time in the forced swim test and a decreased proportion of central motion distance in the open field test, compared to mice transplanted with bacteria obtained from healthy control subjects [101]. On the other hand, it was demonstrated that chronic stress in laboratory animals can shift the microbial diversity in the gastrointestinal tract [102, 103]. Dysbiosis of the gut microbiome observed in depressed patients can also be caused by treatment with some antidepressant drugs due to their antimicrobial activity [104].

The gut microbiota may influence the functioning of the host organism by modulating innate and adaptive immune responses [105]. Importantly, they can also convert indigestible carbohydrates into short-chain fatty acids (SCFA), which in turn activate specific G protein-coupled fatty acid (FFA) receptors, thereby promoting the secretion of peptide hormones from the enteroendocrine cells [106, 107].

Since brain GLP-1R signaling is to a large extent directly triggered by its peripheral secretion from the intestines via vagal stimulation of the NTS neurons, it seems plausible that proper balance in gut microbiota abundance and diversity can influence action of this peptide in the central nervous system. In fact, there are reports which demonstrate that administration of both a probiotic microorganism strain and prebiotics influences GLP-1 release and signaling. In mice, treatment with a probiotic strain of *Bifidobacterium* elevated plasma levels of this hormone [108]. Similar results were also observed in humans. In a study involving healthy non-diabetic subjects, daily intake of a *Lactobacillus reuteri* SD5865 strain for 8 weeks resulted in an increase of GLP-1 secretion in the intestines, which was accompanied by elevated plasma insulin and C-peptide levels [109]. In a clinical study involving patients with type 2 diabetes, an almond-based diet with a low-carbohydrate content, which had been previously shown to possess marked prebiotic properties [110, 111], resulted in augmentation of the number of SCFA-producing bacteria species, such as *Roseburia*, *Ruminococcus* and *Eubacterium*, which led to an increase in fasting blood GLP-1 levels and also promoted antidepressive effects after three months [112].

Many recent studies also demonstrate that GLP-1R agonists display protective effects on the gastrointestinal tract. Treatment with liraglutide was shown to modulate the diversity of the gut microbiota in experiments conducted on animals on a high-fat diet [113, 114]. Similar results were found in a clinical study in patients with T2DM, where administration of liraglutide, but not metformin, increased the relative abundance of *Akkermansia*, which is often associated with the improvement of gastric mucosa and also becomes severely depleted in diabetic patients [115]. Administration of exendin-4 ameliorated stress-induced defecation, decreased visceral pain sensitivity and increased the serum level of anti-inflammatory cytokine: interleukin-13 in the Wistar–Kyoto rat strain, which was considered a model for irritable bowel syndrome [116]. However, this particular rat strain is also widely regarded as a valid model of endogenous depression [117].

## Current clinical evidence for possible antidepressant effects of GLP-1R agonists

Depression is often comorbid with metabolic disorders such as T2DM and obesity and many antidiabetic drugs, which often display neuroprotective and immunomodulatory effect, have demonstrated antidepressant properties in clinical trials. However, in contrast to anti-hyperlycemic agents such as metformin, pioglitazone and peroxisome proliferator-activated receptor gamma (PPARγ) agonists, which were particularly well studied in this matter, very few clinical studies concerning the effects of GLP-1R targeting therapeutics on alleviation of depressive symptoms in humans have been carried out so far [118, 119].

Recent systemic review and meta-analysis demonstrated, that treatment with functional agonists of GLP-1 which included both GLP-1RAs and DPP4 inhibitors resulted in a significant reduction of depression rating scores and tended to be superior over control treatments, which supports the hypothesis of their antidepressant action in humans. These results, however, must be taken into careful consideration, mainly because of a small number of analyzed clinical studies and high between-study heterogeneity [120].

The results of different clinical studies are so far inconclusive and they vary much, depending on experimental design, sample size, health conditions and the chosen rating scale used to asses depressive symptoms in participants. For example, in a prospective study aimed at investigating the influence of GLP-1RA and DPP4 inhibitors on the development of depression in patients with newly diagnosed T2DM, participants receiving incretin-based therapies displayed marked reduction of depressive symptoms after one year, when compared to the control group. These results furthermore correlated with the reduction of CRP levels, which may suggest that anti-inflammatory action may be an important mechanism of the antidepressant effect of incretin-based drugs [121]. In a small-scale four-week long, open-label study involving 14 individuals diagnosed with major depressive disorder and bipolar disorder, which also displayed impaired executive function, treatment with liraglutide significantly reduced Hamilton Depression Rating Scale (HDRS) scores and improved executive function, however, at the same time it did not improve cognitive function in patients [122]. Conversely, in a randomized controlled trial involving adults with T2DM, administration of liraglutide for 26 weeks in addition to standard therapy with insulin, although significantly decreased body weight and levels of glycated hemoglobin HbA1c in patients, but at the same time failed to reduce Beck’s Depression Inventory (BDI) scores (de Witt 2014). There are also few studies concerning the safety of GLP-1RA implementation in neuropsychiatric diseases. In a pooled analysis of phase 2 and 3a clinical trials of liraglutide for weight management, when administered once daily at a dose of 3.0 mg caused no major concerns regarding its neuropsychiatric safety [123].

In summary, there are evidence, that treatment with GLP-1RAs may prove useful in the treatment of depressive disorders, however, the number of reliable clinical reports concerning this matter remains very low at this point. Therefore, further clinical studies are needed to verify whether therapies GLP-1 functional agonists could be used as supportive therapy to currently available antidepressant drugs or whether these compounds would have a beneficial effect in patients suffering from treatment-resistant depression [120].

## Concluding remarks

In this review, we have presented some premises surrounding the potential of GLP-1 receptor agonists for exerting antidepressant effects by outlining their impact on some key processes, which are known to be involved in the pathogenesis of this mental illness. First and foremost, GLP-1 mediated signaling in the brain has a well-established neuroprotective effect, which has been repeatedly proven to enhance memory and cognitive functions, improve neurogenesis and synaptic functions, as well as restore balance in neurotransmission, i.e. processes that are severely impaired in depressed patients. The results of some of the studies we have discussed also seem to indicate that administration of GLP-1RAs may directly exert an antidepressant effect in experimental animals.

Moreover, taking into account numerous clinical studies and experimental data showing that the pathogenesis of depression is not limited to the brain, but may also result from an impaired cross-talk between the central nervous system and other systems in the body, particularly the immune, endocrine and digestive systems, therapeutic GLP-1 analogs such as exendin-4 or liraglutide can also normalize their functioning in many cases, thus preventing the occurrence of maladaptive brain changes associated with depressive symptoms. For example, GLP-1As frequently display immunomodulatory properties, which appears to be noteworthy, since chronic inflammatory processes in the brain are often associated with a depressive mood in patients. The clear influence of therapeutic GLP-1 analogs on the physiology of the digestive system as well as the stability and diversity of the gut microbiota deserves special attention because disorders of the gut-brain axis homeostasis also appear to be an important, although so far somewhat understudied factor, which may lead to the development of depression. Finally, since endogenous GLP-1 is involved in the regulation of energy metabolism both in the periphery as well as in the brain, compounds acting as GLP-1R agonists can also normalize mitochondrial function, which also appears to be repeatedly disrupted in the depressive brain and thus possibly exacerbate neuroprotective effects, e.g. by diminishing the damage caused by oxidative stress.

Although GLP-1RAs seem to have a beneficial influence in reversing many maladaptive brain changes relevant to depression, it should be remembered that at the same time, they are also known to mediate some unfavorable processes such as activation of the HPA axis and intensification of anxiety-like behavior in some animal studies. Even though this phenomenon has not been fully understood in the context of the possible exacerbation of pro-depressive changes by GLP-1RA administration, it certainly requires careful investigation before the potential introduction of these compounds to the clinic for the treatment of mood disorders in the future.

In summary, the GLP-1 receptor remains an interesting and noteworthy target for the pharmacotherapy of central nervous system diseases, not only those related to neurodegeneration and substance abuse, but GLP-1RA drugs may also prove effective as adjunctive therapies in the treatment of mood disorders such as depression. Moreover, due to the peripheral action of GLP-1, their use in depressed patients may also prevent the development of metabolic diseases, such as diabetes and obesity, which are often associated with a depressive disorder.

## Abbreviations

5-HIAA: 5-Hydroxyindoleacetic acid
5-HT: Serotonin (5-hydroxytryptamine)
BBB: Blood–brain barrier
BDNF: Brain-derived neurotrophic factor
cAMP: Cyclic adenosine monophosphate
CRH: Corticotropin-releasing hormone
CRP: C-reactive protein
DPP-4: Dipeptidyl peptidase-4
GABA: Gamma aminobutyric acid
GLP-1: Glucagon-like peptide-1
GLP-1R: Glucagon-like-1 peptide receptor
GLP-1RA: Glucagon-like-1 peptide receptor agonist
HPA: Hypothalamic–pituitary–adrenal axis
*Icv*: Intracerebroventricular
*Ip*: Intraperitoneal
IL-1β: Interleukin-1 beta
IL-6: Interleukin-6
NF-κB: Nuclear factor kappa-light-chain-enhancer of activated B cells
NTS: Nucleus tractus solitarius
PKA: Protein kinase A
PPG: Preproglucagon
PVN: Paraventricular nucleus
SCFA: Short chain fatty acid
T2DM: Type 2 diabetes mellitus
TNF-α: Tumor necrosis factor alpha
VTA: Ventral tegmental area
